# Integrating gene and lncRNA expression to infer subpathway activity for tumor analyses

**DOI:** 10.18632/oncotarget.22811

**Published:** 2017-11-30

**Authors:** Chunlong Zhang, Yanjun Xu, Haixiu Yang, Yingqi Xu, Qun Dong, Siyao Liu, Tan Wu, Yunpeng Zhang

**Affiliations:** ^1^ College of Bioinformatics Science and Technology, Harbin Medical University, Harbin 150081, China

**Keywords:** subpathway, lncRNA

## Abstract

LncRNAs acting as miRNA sponges to indirectly regulate mRNAs is a novel layer of gene regulation, therefore, it is necessary to integrate lncRNA and gene levels for interpreting tumor biological mechanism. In this study, we developed a lncRNA-gene integrated strategy to infer functional activities for tumor analyses at the subpathway level. In this strategy, we reconstructed subpathway graphs by embedding lncRNA components and considered the expression levels of both genes and lncRNAs to infer subpathway activities for each tumor sample. And the activities were applied to three aspects of tumor analyses; First, the subpathway activities across tumor samples of five tumor types were analyzed, and it was observed that the samples with consistent subpathway activities were derived from the same or similar tumor types. Also, the subpathway activities could stratify samples into several subtypes which has different clinical characterization, e.g. survival status. Second, the subpathway activities between tumor and normal samples were analyzed, and the comparative results showed that subpathway activities displayed more specificities than entire pathway activities. Finally, based on the subpathway activities, we identified prognostic subpathways for lung cancer. Our subpathway-based signatures shared significant overlap with enrichment analysis results and displayed predictive power in the independent testing sets. In conclusion, our integrated strategy provided a framework to infer subpathway activities for tumor analyses and identify subpathway signatures for clinical use.

## INTRODUCTION

Long non-coding RNAs (lncRNAs) are found to play key roles in human diseases by mediating a wide range of biological processes, including cell differentiation [1], immune response [2], genomic imprinting [3], and chromatin modification [4]. Especially, recent studies have demonstrated that lncRNAs could act as miRNA sponges to competitively regulate messenger RNA (mRNA) expressions by sharing common miRNA binding sites with mRNAs, which is a novel layer of gene regulation [5, 6]. And, the dys-regulation of lncRNA expressions could further affect biological pathways by competitively regulating mRNAs. For example, the study of Wang *et al.* found that lncRNAs promoted tumorigenesis, invasion, and metastasis by regulating core genes involved in transforming growth factor-β signaling pathway [7]. In another finding, lncRNA CASC11 interacted with gene hnRNP-K and activated Wnt/β-catenin pathway to promote colorectal cancer growth and metastasis [8]. Therefore, it was necessary to perform the pathway activity analyses by considering lncRNA expressions.

Numerous methods have been recently developed to analyze tumor biological mechanisms at the function or pathway level. The pathway signatures that represent sets of gene units with consistent functional roles could display a more robust performance than gene signatures. As a major reason, the function-level analyses could reduce the dimensions of high-throughput data sets, which have more variables than sample number [9, 10]. Ooi *et al.* developed an approach to connecting pathways and tumor profiles, and further identified clinical relevant pathway signatures for gastric cancer [11]. Moreover, another method named FAIME was developed to generate functional signatures for tumor analyses; the FAIME calculated functional activities using rank-weighted gene expressions derived from individual sample [12]. Recently, more and more studies tried to identify dysregulated pathways in kinds of human diseases [13, 14]. However, most current methods performed the function- or pathway-level analyses only based on gene expressions, and ignored the regulatory roles of lncRNAs.

Based on pathway topology information, the subpathway concept was proposed in our previous study. In addition, it has been confirmed that key local subpathways, rather than entire pathway, were more subtly explainable to the etiology of diseases [15, 16]. Containing smaller number of components, the subpathway reflects more detailed functional descriptions and thus interprets disease biological mechanisms at a more precise level. The subpathways have been implicated in detecting multiple mechanisms, including drug action [17] and miRNA regulation [18] analyses. Recently, we identified prognostic signatures for lung cancer patients at the subpathway level and verified the subpathway signatures’ predictive power using independent data sets [19]. So, there is no doubt that subpathway-based analysis is necessary to be considered for precise tumor analyses.

Recently, we developed a novel method named subpathway-LNCE to identify dysfunctional subpathways, which were competitively regulated by lncRNAs [20]. In this study, we firstly converted signaling pathways from Kyoto Encyclopedia of Genes and Genomes (KEGG) into undirected graphs with genes as nodes, and reconstructed the pathway graphs based on lncRNA-mRNA regulations, which were identified by simultaneously considering lncRNA-mRNA co-expression relationship and shared miRNA number. Then, interesting lncRNAs and genes were mapped into the reconstructed graphs, and key subpathways were located using “lenient distance” similarity method [16]. Finally, these key subpathways were evaluated using the Wallenius approximation [21] and the significant subpathways were identified. In the result, we applied subpathway-LNCE method into multiple types of tumors and demonstrated that this method was effective to identify risk subpathways. Furthermore, we confirmed the reliability of corresponding subpathway results using independent data sets. Although the regulatory roles of lncRNAs have been applied into risk subpathway identification, the involvement of lncRNAs in inferring subpathway activities was seldom considered.

We hypothesized the functional activities could be accessible from the expression levels of lncRNAs and genes at the subpathway level. In this study, we developed a novel strategy to infer subpathway activities by integrated analysis of gene and lncRNA expressions. We then applied the subpathway activities into three aspects of tumor analyses, including difference analyses across multiple tumor types, difference analyses between normal and tumor samples, and cancer prognosis analysis. Through these analyses, we found that the subpathway activities could distinguish different samples derived from five tumor types, as well as samples between tumor and normal conditions. Moreover, it was shown that subpathway results displayed more specificities than entire pathway results. Based on the subpathway activities, the prognostic signatures were identified for two lung cancer sub-types, and displayed biological meanings and robust predictive power in independent testing sets. In summary, our lncRNA-gene integrated strategy provided a framework to infer subpathway activities for tumor analyses, and further identify subpathway signatures for tumor patient prognostic implications.

## RESULTS

### Infer subpathway activities by integrating gene and lncRNA expressions

It was necessary to simultaneously consider the expression levels of genes and lncRNAs to perform the pathway analyses. In addition, the subpathway displayed advantages over entire pathway with respect to reflecting more detailed functional descriptions. To resolve these issues, we developed a framework to infer subpathway activities by integrated analysis of gene and lncRNA expressions. As shown in Supplementary Figure 1, we performed this framework at two steps as follows:

i) Reconstruct the subpathway graphs by embedding lncRNAs. We first obtained all the biological pathways from KEGG database and converted these pathways into undirected graphs with genes as nodes. Then, the gene-based subpathways were identified using our previously developed R package [16]. And we further embedded lncRNA components into the gene-based subpathways based on the lncRNA-gene regulations from the study of Shi *et al.* [20] (see Material and Methods). After this step, a total of 1644 subpathways were obtained, and most pathways contained less than 5 subpathways and the path: 05200 (pathways in cancer) contained 60 subpathways (see Supplementary Figure 2A). As shown in Supplementary Figure 2B, subpathways contained an average of 20.2 genes and 26.6 lncRNAs, which were smaller than the entire pathway scale. The detailed gene and lncRNA components within subpathway graphs were provided in Supplementary Table 1.

ii) Calculate the subpathway activity and form subpathway profile. We calculated the activities of all subpathways for each sample (tumor or normal) based on gene and lncRNA expression data sets (see Materials and Methods). For a group of samples, a subpathway profile with all subpathways as rows and samples as columns were further formed. And the activities within profile showed the subpathways conditions reflected by the expression levels of both lncRNAs and genes involved.

### Tumor analyses 1: subpathway activities across 5 tumor types

**Figure 1:**
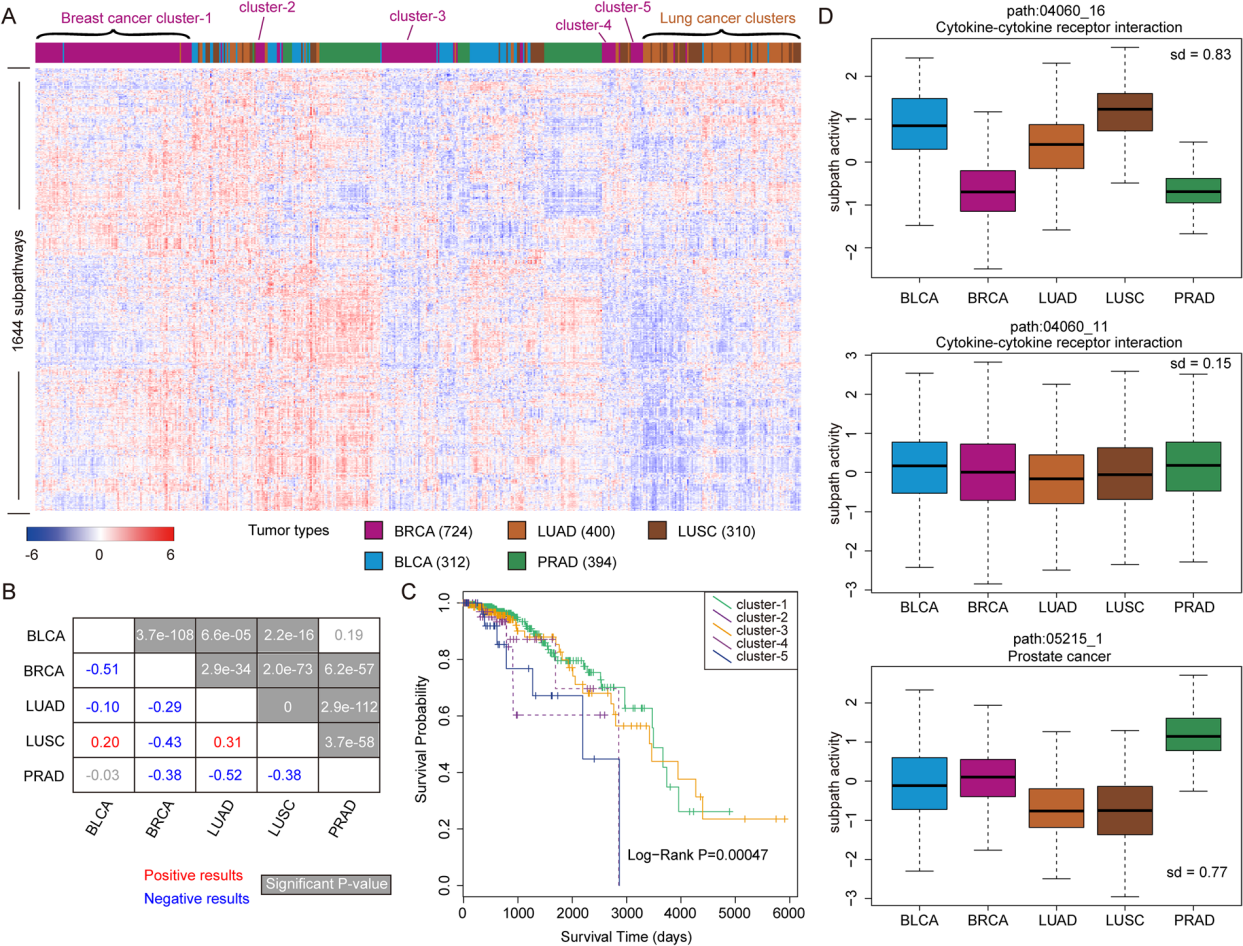
The analyses of subpathway activities across samples from five tumor types **(A)** The clustering results of subpathway matrix with 1644 subpathways as rows and 2140 samples from five tumor types as columns. Some sample clusters are shown as examples, and different colors correspond to each tumor type. **(B)** The correlations between tumor types based on the subpathway activities, and the correlation value is calculated using Pearson method. The red number indicates the positive correlation and the green number indicates negative correlation. The significant results with *P*-values are showed at grey background. **(C)** The Kaplan-Meier survival analyses for samples from five breast cancer clusters (in Figure 1A), and *P*-value is calculated using the Log-rank test. **(D)** Some subpathway examples including path:04060_16, path:04060_11, and path:05215_1 for subpathway activity analyses across five tumor types. The standard deviation of mean subpathway activity among these five tumor types is respectively calculated.

Based on the subpathway activities calculated by integrating gene and lncRNA expressions, we first analyzed the activity difference across tumor types. In this study, we obtained gene and lncRNA expression data sets of totally five tumor types from TCGA database. As detailed described in Material and Methods, the activities of all subpathways were calculated for each tumor sample, and a subpathway profile with 1644 subpathways as rows and 2140 tumor samples as columns was generated. Next, we performed clustering analyses to show the differentiation of subpathway activities among tumor types. As shown in Figure 1A, the samples from the same or similar tumor types displayed a trend to be clustered together. For example, the samples from lung adenocarcinoma and lung squamous cell carcinoma were clustered together to form a cluster, showing that the tumor samples from the same tissue origin displayed consistent subpathway activities. Furthermore, we performed a comparative analyses for subpathway activities between tumor types. For each tumor type, we calculated average subpathway activity based on all tumor samples as the tumor specific subpathway activities. And then, the Pearson correlation of average subpathway activities between five tumor types were calculated. As shown in Figure 1B, most tumor types shared negative subpathway activities with each other, for example, the BLCA displayed negative correlation with BRCA (Pearson value = -0.51, *P*-value = 3.7e-108). And, the LUAD and LUSC displayed positive correlation with Pearson value = 0.31, which further confirmed that consistent subpathway activities were involved in the tumor samples with the same tissue origin.

On the other hand, separate clusters were also formed for the samples from the same tumor type, for example, five breast cancer clusters (from cluster-1 to cluster-5) were separated. As a probable reason, the samples of these five breast cancer clusters might display different biological characterizations. So, we further analyzed whether there existed clinical outcome difference among samples from the above clusters. As shown in Figure 1C, the samples from cluster-1 and cluster-3 displayed the best clinical outcomes, while the samples from cluster-5 displayed the poorest clinical outcomes. And there was significant survival difference (*P*-value = 0.00047) among the five clusters by log-rank test, also indicating the performance of subpathway activities to distinguish patient’s clinical characterization.

We also analyzed the activity difference of specific subpathways across five tumor types. As shown in Figure 1D, path:04060_16 from the Cytokine-cytokine receptor interaction displayed the highest activity difference. In detail, this subpathway displayed low activities in BRCA and PRAD, and displayed high activities in other three tumor types. In the meanwhile, another subpathway (path:04060_11) from the same entire pathway did not display the difference, which reflected the specificity of subpathway results. In addition, we observed that the path:05215_1 from Prostate cancer displayed significantly higher activity in PRAD than other tumor types.

### Tumor analyses 2: subpathway activities between normal and tumor samples

To test whether the subpathway activities could distinguish normal and tumor samples, we obtained cancer pathways from KEGG database, which corresponded to different tumor types. For example, Path: 05219 (Bladder cancer) corresponded to BLCA, Path: 05223 (Non-small cell lung cancer) corresponded to LUAD and LUSC, and Path: 05215 (Prostate cancer) corresponded to PRAD. For performing the comparison between subpathways and entire pathways, we also reconstructed pathway graphs by embedding lncRNA components, which was similar as the procedure for subpathway graphs. Then, we calculated the subpathway and pathway activities for all normal and tumor samples for some certain tumor type, based on the reconstructed subpathway graphs and entire pathway graphs. And the activity between normal and tumor samples was compared using the Wilcoxon rank sum test. Take the Path:05219 (Bladder cancer) as an example, entire pathway and six subpathway graphs were reconstructed, and the activities were both calculated at the pathway and subpathway levels. Using TCGA BLCA data set, we could compare the performance of entire pathway and subpathways in distinguishing normal and tumor samples.

**Figure 2:**
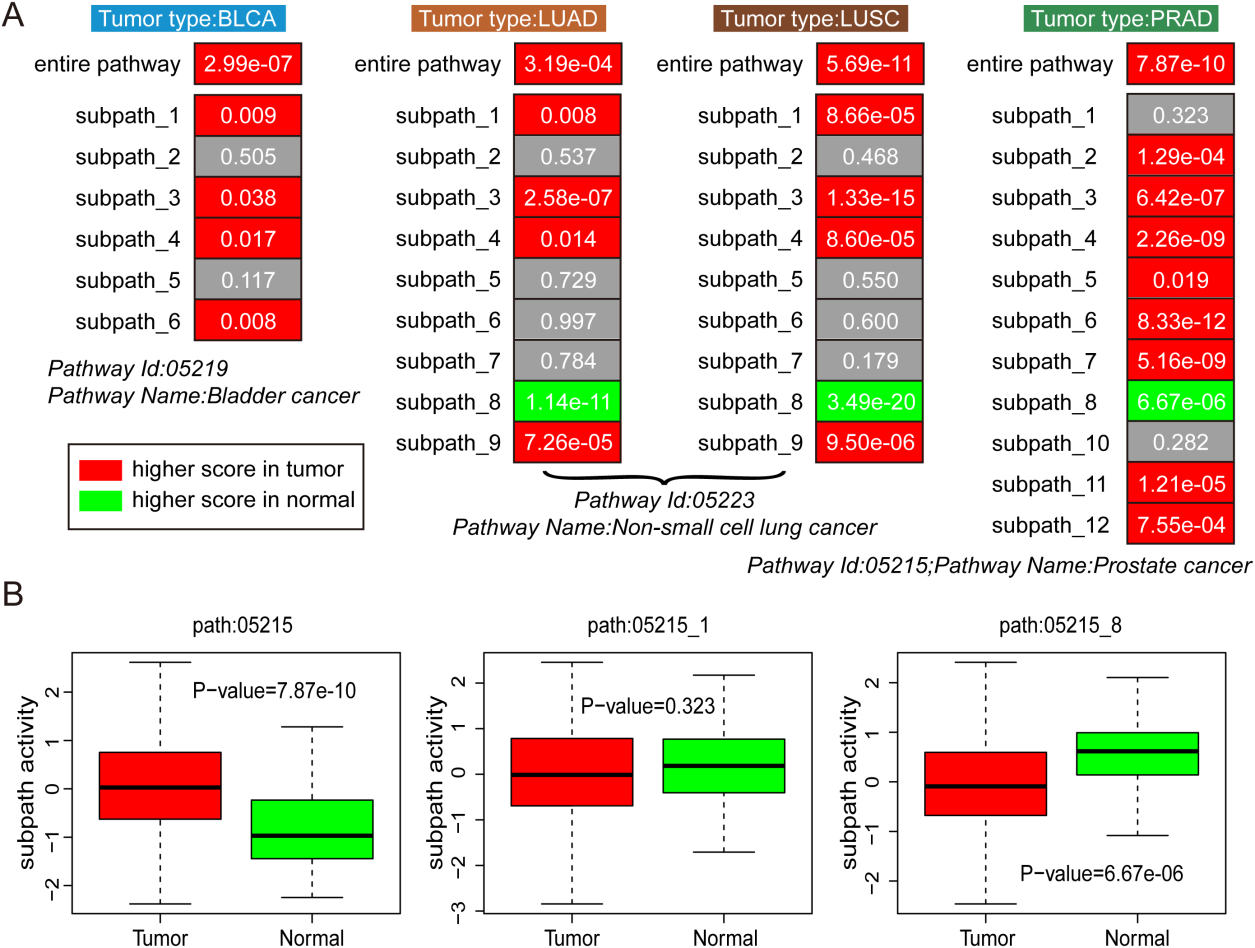
The analyses of subpathway activities between tumor and normal samples **(A)** The performance of entire pathway and subpathway activities in distinguishing tumor and normal samples for four tumor types, including BLCA, LUAD, LUSC, and PRAD. The grey color indicates non-significant results, the red color indicates higher activity score in tumor samples, and the green color indicates higher activity score in normal samples. *P*-value is calculated using the Wilcoxon rank sum test. **(B)** Path: 05215 for PRAD type as an example.

As shown in Figure 2A, entire pathways displayed higher activities in tumor samples than normal samples, with *P*-value = 2.99e-07 in BLCA, *P*-value = 3.19e-04 in LUAD, *P*-value = 5.69e-11 in LUSC, and *P*-value = 7.87e-10 in PRAD. At the subpathway level, most subpathways displayed consistent activities as corresponding entire pathway, higher activity in tumor samples than normal samples. In addition, the path:05223_8 and path:05215_8 displayed opposite subpathway patterns with higher activities in normal samples than tumor samples, showing that novel biological patterns were observed at the subpathway levels. In conclusion, all these findings confirmed that both pathway and subpathway activities could distinguish tumor samples from normal samples, and subpathway analyses displayed more specificity.

### Tumor analyses 3: subpathway activities for tumor prognoses

As shown in the first tumor analyses, the samples from LUAD and LUSC displayed similar subpathway activities, moreover, the patients with non-small cell lung cancer (NSCLC) often had poor prognoses [22], and LUAD and LUSC were major types of NSCLC. Therefore, we applied the subpathway activities into the tumor prognoses and respectively identified prognostic subpathways for LUAD and LUSC risk classifications. For the TCGA LUAD (or LUSC) data set, we first randomly divided it into a training set and a testing set, which contained the same sample number. Based on the training set, we then calculated the subpathway activities for all 1644 subpathways and identified prognostic subpathways using the univariate Cox analyses. Also, we performed a comparison between the prognostic subpathways we identified and the results from traditional hypergeometric enrichment method (see Material and Methods). Finally, we analyzed the survival predictive performance of our prognostic subpathways using corresponding independent testing set.

**Figure 3:**
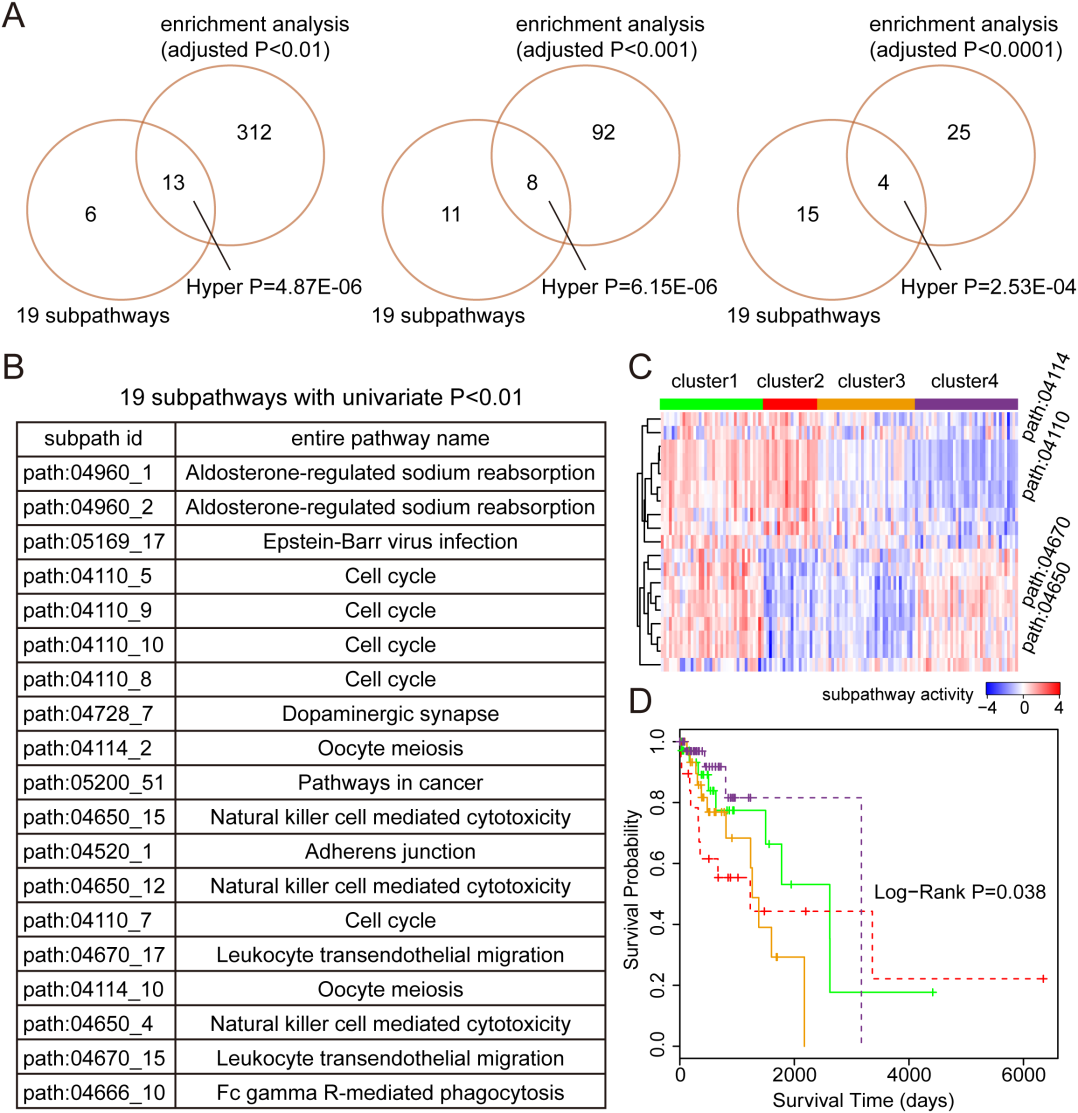
The prognostic analyses based on subpathway activities in LUAD **(A)** The comparison between 19 prognostic subpathways identified by univariate Cox based on subpathway activities and the subpathways identified by enrichment analyses with different adjusted *P*-value cut-offs (0.01, 0.001, and 0.0001). The comparative *P*-values are calculated using the hypergeometric test. **(B)** The subpathway id and entire pathway name of these 19 prognostic subpathways. **(C)** K-mean clustering representation based on subpathway activities using the testing set. The columns represent samples and rows represent the 19 subpathways. The red color indicates high subpathway activity, whereas the green color indicates low activity. **(D)** The Kaplan-Meier analyses of clinical outcome between samples from four risk clusters (in Figure 3C), and *P*-value is calculated by the log-rank test.

For LUAD, a total of 19 subpathways were identified as prognostic signatures with Univariate Cox *P*-value < 0.01, and these subpathways were derived from 10 entire pathways (see Figure 3). As a result of comparison, these 19 prognostic subpathways shared significant overlap with the subpathway identified by enrichment analyses. In detail, 13, 8, and 4 subpathways were commonly identified with enrichment analysis *P*-value < 0.01, 0.001, and 0.0001. It was shown that our method based on the subpathway activities displayed consistent results with traditional pathway identification method. To test the survival performance of the 19-subpathway signatures, we performed K-mean clustering to achieve tumor sample classification using the testing set. As a result, four risk sample groups were formed in the clustering analyses and K-M survival analysis was applied to evaluate the survival difference between different risk groups. As shown in Figure 3C and 3D, the samples from cluster 4 with low subpathway activities from cell cycle pathways (path:04114 and path:04110) as well as high subpathway activities from immune pathways (path:04670 and path:04650) displayed the best prognoses, whereas, the samples from cluster 2 with high subpathway activities from cell cycle pathways (path:04114 and path:04110) as well as low subpathway activities from immune pathways (path:04670 and path:04650) displayed the poorest prognoses. And the log-rank test showed that there was significant difference in survival time between these four risk groups (*P*-value = 0.038).

**Figure 4:**
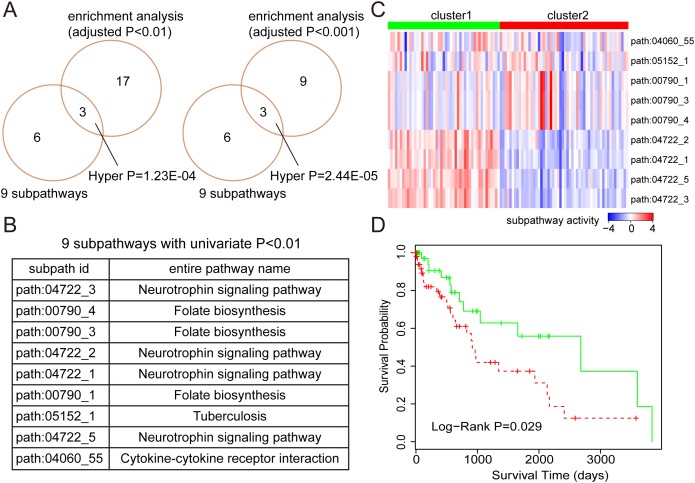
The prognostic analyses based on subpathway activities in LUSC **(A)** The comparison between 9 prognostic subpathways identified by univariate Cox based on subpathway activities and the subpathways identified by enrichment analyses with different adjusted *P*-value cut-offs (0.01 and 0.001). The comparative *P*-values are calculated using the hypergeometric test. **(B)** The subpathway id and entire pathway name of these 9 prognostic subpathways. **(C)** K-mean clustering representation based on subpathway activities using the testing set. The columns represent samples and rows represent the 9 subpathways. The red color indicates high subpathway activity, whereas the green color indicates low activity. **(D)** The Kaplan-Meier analyses of clinical outcome between samples from two risk clusters (in Figure 4C), and *P*-value is calculated by the log-rank test.

For LUSC, 9 subpathways were identified as prognostic signatures with Univariate Cox *P*-value < 0.01. And, we also observed that there was significant number of common subpathways between these 9 subpathways and subpathways identified by enrichment analyses (see Figure 4A). Similarly, we tested the predictive power of these 9 subpathways using corresponding testing set. As shown in Figure 4C and 4D, the samples with high subpathway activities from neurotrophin signaling pathway as well as low subpathway activities from folate biosynthesis displayed good prognoses, whereas, the samples with opposite subpathway activity patterns displayed poor prognoses. And log-rank test showed that there was significant difference in survival time between these two risk groups (*P*-value = 0.029).

## DISCUSSION

The tumor function-based characterization was not well understood, and lncRNA expressions should be considered in inferring functional activities at the subpathway level. In this study, we developed a novel strategy to infer subpathway activities by considering the expression levels of both genes and lncRNAs. And the subpathway activities were applied into three tumor analyses. In first tumor analyses, we found that the subpathway activities could distinguish tumor samples from different tumor types, and the samples with the same histological origin were clustered together. In second tumor analyses, the subpathway activities could distinguish tumor samples from normal samples, and subpathway-based results were proved to outperform the entire pathway-based results. Finally, we respectively identified two prognostic signatures for LUAD and LUSC based on the subpathway activities, and verified the predictive power of these signatures using testing set.

LncRNAs functioned as competitors of mRNAs by binding miRNAs, thereby competitively regulating mRNA expression levels [5, 6, 23]. The sponge features of lncRNAs could competitively regulate genes in biological pathway and thus played critical roles in tumor initiation and progression [23]. PTENP1 was a PTEN pseudogene and contained seed sequences for PTEN-targeting miRNAs [24, 25]. The study of Wang *et al.* found that lincRNA-RoR played roles as miRNA sponge to regulate Oct4 and Sox2, and then mediated differentiation processes [26]. Moreover, as an oncogenic lncRNA in multiple cancers, H19 functioned as miRNA sponge to lead to the de-repression of ZEB1 and ZEB2 genes in the mesenchymal cells [23]. In this study, the lncRNA-gene interactions were obtained from our previous study [20], which considered both sequence-based and expression-based associations. In the sequence-based associations, the lncRNA-gene pairs were evaluated using both hypergeometric test *P*-value and Jaccard Coefficient indexes. In the expression-based associations, the lncRNA-gene pairs which were co-expressed in at least 3 of 28 RNA-seq data sets were required, which were also utilized in other studies [27, 28].

The gene components of distinct signatures usually displayed no significant overlap, even though these signatures exhibited efficient power [29]. Therefore, it was necessary to perform the function-level analyses for interpreting tumor biological mechanism. In addition, concentrating more attention on subpathways rather than entire pathways might be more biologically meaningful. Recently, our team has performed a series of researches to explore the applications of subpathways, including disease etiology, drug action and miRNA regulation [17, 18, 30]. In this study, we performed a comparison between subpathway-based and entire pathway-based results. As shown in Figure 2B, subpathways exhibited more informative than entire pathways to distinguish tumor and normal samples. Different subpathways derived from the same pathway gave opposite trends in tumor and normal samples; the subpath_8 from the path: 05215 (Prostate cancer) displayed high activities in PRAD normal samples, whereas the entire pathway and other subpathways displayed high activities in PRAD tumor samples. The high-resolution subpathway activities could provide novel insights into tumor molecular mechanisms.

As an important application, we respectively identified prognostic subpathways for LUAD and LUSC based on the subpathway activities. For LUAD, the subpathways derived from Cell cycle, Oocyte meiosis, Pathways in cancer, Leukocyte transendothelial migration, and Natural killer cell mediated cytotoxicity were identified. And different activity patterns within these subpathways were observed in four LUAD clusters. The samples in cluster 2 displayed high activities for cell cycle subpathways and low activities for immune-related subpathways, whereas the samples in cluster 4 displayed opposite activity patterns with cluster 2. Also, the survival analyses showed that there was significantly difference in survival time between these clusters. For LUSC, 9 subpathways derived from Neurotrophin signaling pathway, Folate biosynthesis, and Cytokine-cytokine receptor interaction were identified, and also displayed predictive power, which indicated that these subpathways might be involved in LUSC formation and progression.

The data sets from TCGA database simultaneously detected the expression levels of genes and lncRNAs, and provided reliable resource to perform integrated analysis. Based on the subpathway activities calculated, we performed a series of applications including the characterization across tumor types, the characterization between normal and tumor samples, and tumor patient’s prognoses. This integrated strategy provided a framework for inferring subpathway activities, which could be applied on other human complicated diseases.

## MATERIALS AND METHODS

### Data sets from TCGA

We obtained RNA level 3 expression data of five tumor types from The Cancer Genome Atlas (TCGA) database (version April, 2015) through the Data portal. These five tumor types included bladder urothelial carcinoma (BLCA), breast invasive carcinoma (BRCA), lung adenocarcinoma (LUAD), lung squamous carcinoma (LUSC), and prostate adenocarcinoma (PRAD). For each tumor type, we extracted the expression data sets of lncRNAs and genes from the raw read counts of each exon, which were obtained from exon quantification files (RNASeqV2). Then, we recalculated the RPKM expression values of lncRNAs and mRNAs for each sample, and the detailed calculation process was described in our previous study [31]. Both tumor and normal samples were considered in the tumor analyses. For BRCA, LUAD and LUSC types, we also obtained the clinical information for patient prognosis analyses.

### LncRNA-gene regulations

The competitively lncRNA-gene regulations were obtained from our previous study in which we reconstructed lncRNA-mediated pathway graphs [20]. In brief, we firstly predicted the miRNA-lncRNA and miRNA-gene interactions based on six miRNA target prediction methods and the Argonaute-CLIP data. And, the lncRNA-gene pairs that shared large number of miRNAs using two indexes (hypergeometric *P*-value < 0.05 and rank of Jaccard Coefficient in top 20%) were identified as candidate lncRNA-gene regulations. Then, the co-expression relationship between candidate lncRNA-gene pairs in 28 RNA-seq data sets were further considered. The details were described in the study of Shi *et al.* [20]. Finally, we got 6722 non-redundant lncRNA-gene regulations including 798 lncRNAs and 1527 genes for further analyses.

### Reconstruction of subpathway graphs by embedding lncRNAs

We reconstructed subpathway graphs by embedding lncRNA components if lncRNAs had regulatory relationships with genes within the subpathway. The detailed processes were described below. First, we extracted all the biological pathways from KEGG database, and converted them into undirected graphs using our previously developed R package [16]. In these pathway graphs, nodes represent genes and edges between two nodes represent that these genes interacted with each other in this pathway. Then, we used K-clique method to identify subpathways based on the shortest paths among genes in each pathway. In this process, the distance between any two gene nodes within subpathway was no larger than *k*, and the default value (*k*=3) was used. Finally, we determined whether one lncRNA was embedded in the subpathway based on competitively lncRNA-gene regulations obtained from our previous study [20]. And the lncRNAs which regulated at least one gene within the subpathway were embedded into this subpathway. To reduce bias of small scale, subpathway graphs with less than two lncRNA or three genes were not considered in further analyses. And the reconstructed subpathway graphs, which incorporated both lncRNA and gene nodes, were used for calculating subpathway activities.

### Calculation of subpathway activities

The activity for each subpathway was calculated by simultaneously considering the expression levels of genes and lncRNAs within this subpathway using modified FAIME method [12]. First, we merged the gene and lncRNA expression profiles to form a matrix with genes and lncRNAs as rows and common samples as columns. Then, we sorted all expressed genes and lncRNAs $\left(N_{g+\ln\;c}\right)$ of each sample in an ascending order according to their expression levels, and then weights (w) of the ordered genes and lncRNAs $\left(w_{g+\ln\;c,s}\right)$ were calculated as follows:

$$w_{g+\ln\;c,s}=\left(r_{g+\ln\;c,s}\right)\cdot \left(e^{\frac{r_{g+\ln\;c,s}}{\left|N_{g+\ln\;c}\right|}}\right)$$

Where $r_{g+\ln\;c,s}$ was the rank for each gene and lncRNA in the sample s, and |N| was the total number of genes and lncRNAs in the merged matrix.

For each reconstructed subpathway graph, two sets were defined: ${subpath}_{i}$ in which components satisfied $component\in {subpath}_{i}$ and a complement component-set $\left(N/{subpath}_{i}\right)$ in which components were involved in the merged matrix but not in the subpathway graph. Then, the subPathway Activity (sPA in equations) score was calculated as follows:

$$sPA_{subpath_{i}},s=\frac{1}{\left|{subpath}_{i}\right|}\sum_{g+\ln\;c\in {subpath}_{i}}\left(w_{g+\ln\;c,s}\right)-\frac{1}{\left|N/{subpath}_{i}\right|}\sum_{g+\ln\;c\in N/{subpath}_{i}}\left(w_{g+\ln\;c,s}\right)$$

For comparison, a z-type statistic was used to define normalized subPathway Activity (sPA_norm) as follow:

$$sPA\_norm_{{subpath}_{i},s}=\frac{sPA_{{subpath}_{i},s}-\bar{sPA_{{subpath}_{i}}}}{S\left(sPA_{{subpath}_{i}}\right)}$$

Where $\bar{sPA_{{subpath}_{i}}}$ represented the mean value of subpathway i across all samples and the $S\left(sPA_{{subpath}_{i}}\right)$ represented the standard deviation.

### Hypergeometric enrichment method

For convenience, most researchers treated the gene elements within pathway graphs as independent and applied hypergeometric test to pathway analyses. For a comparison of prognostic subpathways, we also utilized hypergeometric enrichment analyses to perform subpathway identification. In detail, we used univariate Cox method to identify prognostic genes and lncRNAs (*P*-value < 0.05) based on TCGA training sets. Then, the significance *P*-value for each reconstructed subpathway graph was evaluated as follows:

$$P=1-\sum_{k=0}^{r_{g}+r_{\ln\;c}-1}\frac{\left(\begin{array}{c} t_{g}+t_{\ln\;c}\\ k \end{array}\right)\left(\begin{array}{c} m_{g}+m_{\ln\;c}-t_{g}-t_{\ln\;c}\\ n_{g}+n_{\ln\;c}-k \end{array}\right)}{\left(\begin{array}{c} m_{g}+m_{\ln\;c}\\ n_{g}+n_{\ln\;c} \end{array}\right)}$$

where $m_{g}$ $\left(m_{\ln\;c}\right)$ was the number of genes (lncRNAs) in entire genome (lncRNAome), of which $t_{g}$ $\left(t_{\ln\;c}\right)$ genes (lncRNAs) were involved in the reconstructed subpathway graph, and the number of prognostic genes (lncRNAs) was $n_{g}$ $\left(n_{\ln\;c}\right)$, of which $r_{g}$ $\left(r_{\ln\;c}\right)$ genes (lncRNAs) were also involved in the same subpathway. The corrected *P*-values were finally calculated using the Benjamini-Hochberg method.

### Survival analyses

In the survival analyses, we first used K-means clustering method to divide tumor samples into different risk groups. Then, Kaplan-Meier (K-M) analyses were performed to compare the survival differences of the patients in these risk groups. The significance of differences between groups was finally tested using the log-rank test. Moreover, prognostic power of subpathway signatures were also evaluated using univariate Cox analyses. In all these survival analyses, *P*-value < 0.05 was considered as significant.

### Clustering analyses

We performed hierarchical clustering analyses for subpathway activities across 2140 tumor samples of five tumor types. The correlation (uncentered) and complete linkage methods were selected to perform the analyses using Cluster3 software. Finally, the Java TreeView imaging software was used to display the clustering results.

## SUPPLEMENTARY MATERIALS FIGURES AND TABLE




