# Supplementary material for: Integrating gene and lncRNA expression to infer subpathway activity for tumor analyses
Source: Oncotarget. 2017 Nov 30;8(67):111433–43. doi: 10.18632/oncotarget.22811 (PMC5762333; doi:10.18632/oncotarget.22811)
Supplement: Supplementary file 1 [file oncotarget-08-111433-s001.pdf]

Integrating gene and lncRNA expression to infer subpathway activity for tumor analyses

SUPPLEMENTARY MATERIALS

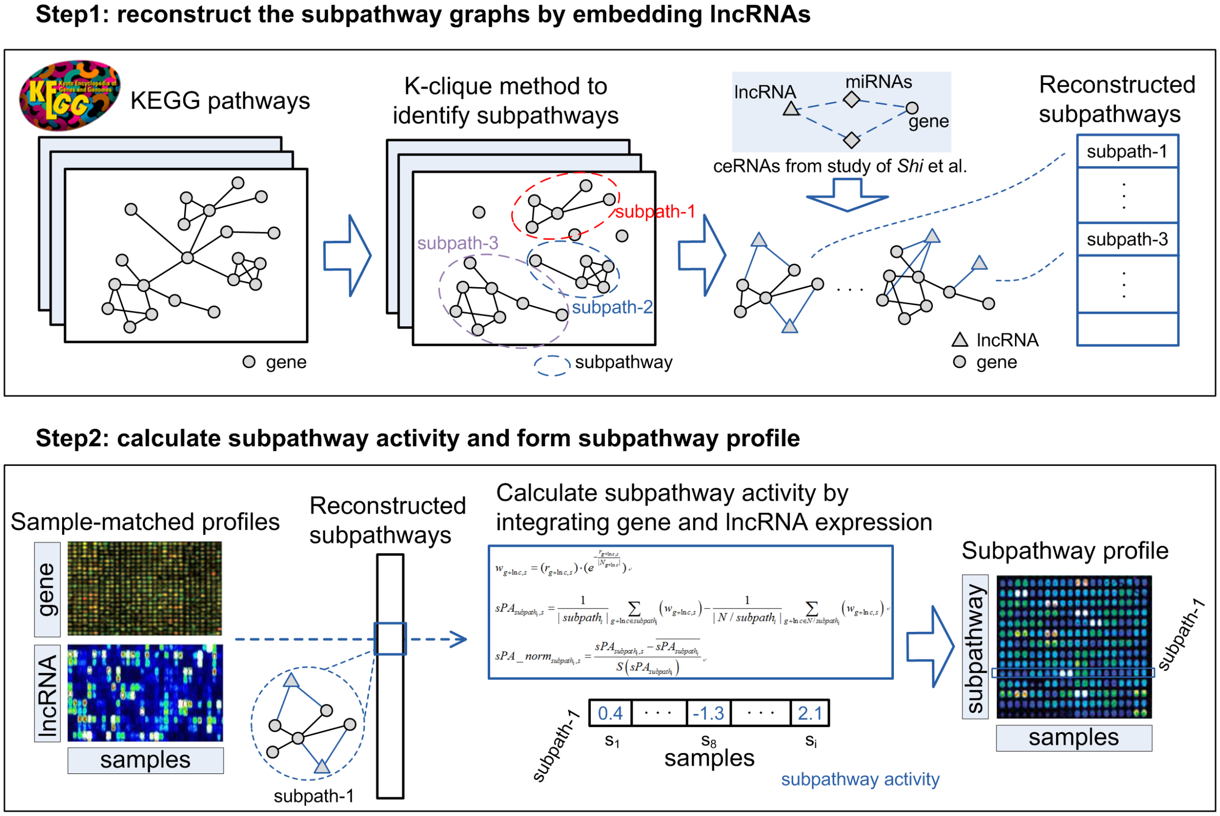

Supplementary Figure 1: The workflow of our integrated lncRNA-gene strategy. Step1: reconstruct the subpathway graphs by embedding lncRNAs. Step2: calculate the subpathway activities and form subpathway profile with subpathways as rows and samples as columns.

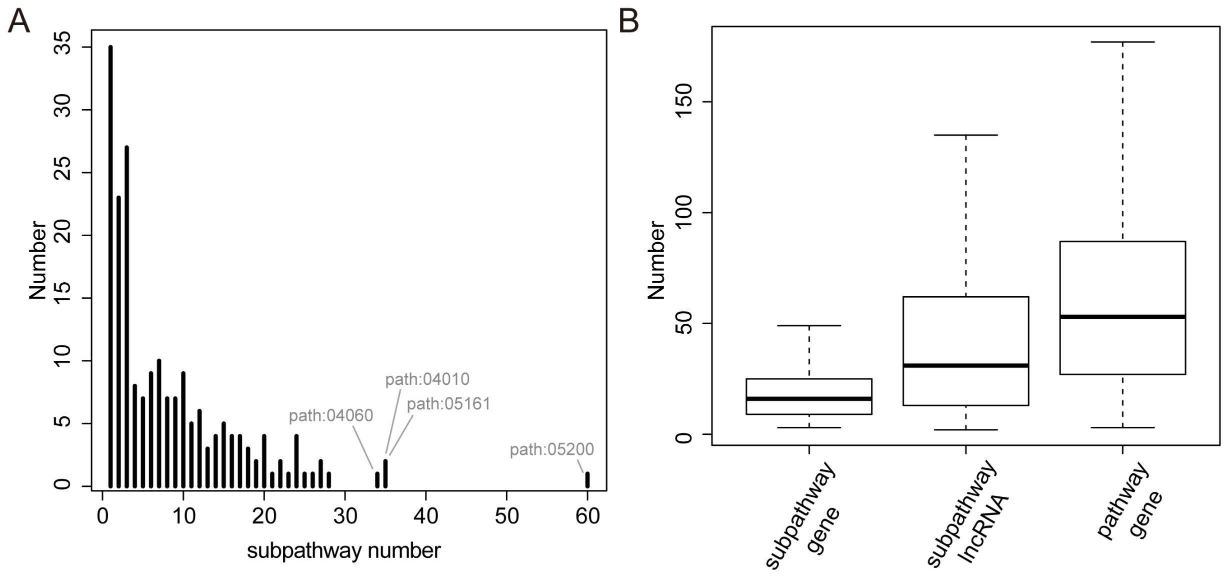

Supplementary Figure 2: The analyses of reconstructed subpathway graphs. **(A)** The number of subpathways within each entire pathway. **(B)** The average number of gene and lncRNA nodes within subpathway graphs, and the average number of gene nodes within pathway graphs.

Supplementary Table 1: The detailed gene and lncRNA components within subpathway graphs

See Supplementary File 1
